# Thiel cadaver eye as a training model for sub-Tenon’s blocks: a feasibility study

**DOI:** 10.1186/s12886-024-03630-3

**Published:** 2024-09-03

**Authors:** Friedrich Lersch, Damian Schnidrig, Susanne Boemke, Valentin Djonov, Damian Jaggi, Florian M. Heussen

**Affiliations:** 1 Department of Anaesthesiology, Inselspital, University Hospital Bern, Bern, 3010 Switzerland; 2 Haag-Streit AG Diagnostics, Köniz, Switzerland; 3 Institute of Anatomy, University of Bern, Bern, Switzerland; 4 Department of Ophthalmology, Inselspital, University Hospital Bern, Bern, Switzerland

**Keywords:** Tenon’ capsule, Local anaesthesia, Cadaver, Simulation training

## Abstract

**Background:**

Regional anaesthesia education, especially for ocular procedures, necessitates reliable surgical training models. While cadaveric models offer anatomical fidelity, conventional embalming methods may compromise tissue integrity. We aimed to assess the effectiveness of Thiel cadavers for training in sub-Tenon’s blocks by evaluating ocular tissues and measuring insertion forces.

**Methods:**

Experimental design, using twenty eyes from ten Thiel cadaver heads. These cadavers were specifically prepared to test the administration of sub-Tenon’s blocks. The research was conducted in a controlled laboratory setting appropriate for handling cadaveric materials and conducting precise measurements. Each cadaver eye underwent an initial ultrasound examination, and its axial length was noted. An intravitreal injection of heptastarch solution followed, to re-establish the eye’s sphericity. After this volume injection, the axial length and intraocular pressure were measured again. Mock sub-Tenon’s blocks were administered in 2 separate quadrants of the eye, with insertion forces measured using a pressure gauge. These were compared to a data set of insertion forces measured in a series of isolated pig’s eyes on which STBs had been performed. Main outcome measurements were macroscopic assessment of the ocular tissue layers and the insertion forces required for the sub-Tenon’s blocks. In a second set of 10 Thiel cadaver heads, 5 ml of sodium chloride were injected as sub-Tenon’s blocks and the emergence of a periocular “T-sign” ascertained and measured by ultrasound.

**Results:**

Four of twenty eyes (20%) retained near-natural sphericity, with the remaining requiring volume injection to approximate physiological shape and pressure. The conjunctiva and Tenon’s layer were intact, and correct cannula placement was achieved in all cases. In 16 of 20 eyes where T-signs could be measured, the median thickness of the T-sign amounted to 2.72 mm (range 1.34 mm–5.28 mm). The average maximum cannula insertion force was 2.92 Newtons. Insertion forces in intact Thiel cadaver heads were consistently higher than in isolated pig’s eyes (3.6 N vs 2.0 N).

**Conclusion:**

These findings suggest that Thiel cadavers are a promising model for training in sub-Tenon’sblocks, despite the challenge of often desiccated and involuted eyes.

**Supplementary Information:**

The online version contains supplementary material available at 10.1186/s12886-024-03630-3.

## Key points

• Thiel cadaver eyes were evaluated as training model for sub-Tenon’s blocks for the first time

• The cadaver eyes were often involuted and required fluid injection to reproduce physiological bulbar tonus

• The ocular tissues and anatomical layers were well preserved

• T-signs as a hallmark of successful sub-Tenon’s block can be ascertained in Thiel cadaver eyes

• Dynamic insertion forces were measured during cannula placement and can be used as reference for future studies comparing haptics of different models by an objective measure-insertion force.

**Supplementary Information:**

The online version contains supplementary material available at 10.1186/s12886-024-03630-3.

## Background

In addition to background knowledge and skill, practise on a suitable and reliable training model would further enhance the learning of regional anaesthesia technique and potentially reduces block-related complications, both for novel beginners and experienced clinicians. This applies to ocular regional anaesthesia training as much as to any other regional block. Non-cadaver models are often inadequate in replicating accurate anatomy and tissue sensations [1] and, while significant, can only cover the fundamental elements of ocular regional anaesthesia [2, 3]. Animal cadaver models can present several simulation issues in themselves [4], whereas human cadaver training is expensive and restricted by availability and stiffening of dead tissue through formalin embalming. Under these circumstances, the use of Thiel cadavers significantly enhances surgical and anaesthetic simulations. Expenses for training on these model often can only be shouldered by tertiary training hospitals or international congresses. The Thiel embalming process effectively conserves tissue without denaturing its proteins, consequently preserving anatomical relationships and maintaining the near-life softness of tissue [5]. Accordingly, anaesthetic societies are increasingly using Thiel cadavers to teach ultrasound-guided regional nerve blocks. Although there is limited literature on Thiel cadaver models for ocular use, they have been utilized for oculoplastic surgery training [6]. To the best of our knowledge, no study has been conducted on the use of sub-Tenon’s blocks in Thiel cadaver eyes.

An essential component of any regional anaesthetic procedure is the application of forces that provide direct haptic feedback about possible breaches in the anatomical layers. This also allows assessing whether the injection needle has been accurately placed. Anaesthesiologists who perform epidural and spinal anaesthesia are familiar with this concept [7, 8]. Extensive evaluation of the forces applied during regional anaesthesia has been conducted to prevent nerve damage caused by needles [9]. Therefore, it is logical to concentrate on this aspect during the training.

The aim of the study was to determine whether human Thiel cadavers could serve as an effective training model for sub-Tenon’s (STB) blocks. This involved assessing the integrity of ocular tissues necessary for the procedure and measuring the forces applied during sub-Tenon’s cannula insertion to establish objective and comparable measurements.

## Methods

In this study, we utilized twenty Thiel-embalmed human eyes in 10 cadaver heads obtained from donors through the Institute of Anatomy, University of Bern, in October 2023. In early April 2024, we performed injecting sub-Tenon’s blocks and assertion of ultrasound T-signs on a second set of Thiel cadaver heads. We measured STB-insertion forces in Thiel cadavers and isolated pig-eyes – a traditional training mode we used for comparison.

### Ethics approval and consent to participate

The use of human cadaveric material adhered to the regulations outlined in the Federal Act on Research involving Human Beings (Human Research Act, HRA) (HRA, 2014) and the Guidelines of the Swiss Academy of Medical Sciences (“Verwendung von Leichen Und Leichenteilen in Der Medizinischen Forschung Sowie Aus-, Weiter- Und Fortbildung”, 2009). Donors provided formal consent for using their body parts post-mortem in research through signed donation forms filed by the Berne university institutional review board (Kantonale Ethikkommission für die Forschung, Bern). According to national legislation of 2009 and 2014 (cited above) ethical approval for this study was not required as it did not involve living patients [10, 11].

The Thiel cadaver heads were prepared by mounting them sequentially onto a wooden frame to align the face and eyes horizontally. Each eye’s tonus and sphericity were assessed, noting whether they were concave (involuted, shrunk) or convex. To standardize this evaluation, an experienced ophthalmologist captured ultrasound images of each eye along the optic nerve axis using a 10 MHz Ultrasound probe in B-scan acquisition mode (Aviso, Quantel Medical). The anteroposterior diameter was measured by approximating the Zero-Signal baseline as the eye’s anterior surface. Heptastarch® solution was then injected into the eye bulb until achieving natural sphericity, with the injected volume recorded. After injection, a second ultrasound image was taken, and the anteroposterior diameter was measured. The ratio between pre-injection and post-injection values was calculated. Two mock sub-Tenon’s blocks were performed on each eye: one in the inferonasal quadrant, the other in the superotemporal quadrant.

### Primary outcome

The primary outcome of the study was to assess the suitability of human Thiel cadavers as a training model for sub-Tenon’s (STB) blocks. This was evaluated by examining the integrity of ocular tissues necessary for successful blocks and measuring the applied forces during sub-Tenon’s cannula insertion. In a second set of Thiel cadaver heads, we ascertained the presence of ultrasound T-signs after injecting 5 ml of sodium chloride into the sub-Tenon’s space.

The secondary outcomes included:

- Evaluation of the distribution of natural sphericity among the Thiel cadaver eyes.
- Assessment of intraocular pressure changes post-heptastarch® injection.
- Analysis of the forces required for sub-Tenon cannula insertion in different eye quadrants.

The intraocular pressure after Heptastarch® injection was initially assessed by digitally evaluating the rigidity and firmness of the eye, and then more accurately with a Schioetz tonometer. The double layer of the bulbar conjunctiva and Tenon’s fascia was opened at two different locations in each eye, inferonasally and superotemporally, using a snipping technique [12] with Moorfields surgical forceps and Westcott scissors. The sclera was then visualized (Fig. 3) before introducing the STB-cannula behind the eye’s equator. The STB-cannula was inserted in its entire length, leaving 1–2 mm visible to avoid inducing pressure spikes by the insertion hub hitting the eye’s surface. The force required to insert the cannula behind the eye’s equator was dynamically measured. The insertion force curve was acquired using a Transmetra KD 24S pressure gauge between a handpiece and blunt TriPort cannula [13]. Maximum insertion force values were extracted from this curve. Differences in mean insertion forces for the inferonasal and supratemporal simulated STBs were tested for equality with a two-signed paired t-test. To compare insertion forces in Thiel cadavers to a commonly used training model, we measured these forces in 22 fresh pig eyes using the same method.

## Results

We performed 40 simulated STBs on 20 Thiel cadaver eyes. In a second set of Thiel cadaver heads we performed STBs injecting 5 ml sodium chloride solution and measured presence and depth of T-signs in mm. To compare our results, we measured STB-insertion forces in Thiel cadavers and isolated pig-eyes.

**Fig. 1:**
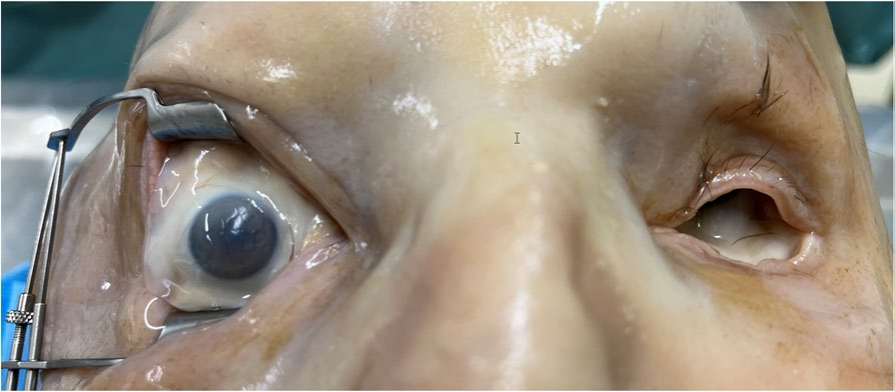
Some eyes retain sphericity, albeit at very low intraocular pressures like the left eye in this cadaver head

Out of 20 eyes examined in the first set of cadaver heads, only 4(20%) maintained some degree of natural sphericity (see Fig. 1), categorized as convex, while the remaining 16 eyes lacked tonus and were completely shrunken or concave. In the second set of 10 cadaver heads, the data of 4 eyes was discarded from the sample due to eye damage (2), or presence of oil after VR surgery (2). Sixteen eyes displayed ultrasound T-signs with a median depth of 2.72 mm (range 1.34 mm–5.28 mm, SD 1.105) (see Fig. 5).

**Fig. 2:**
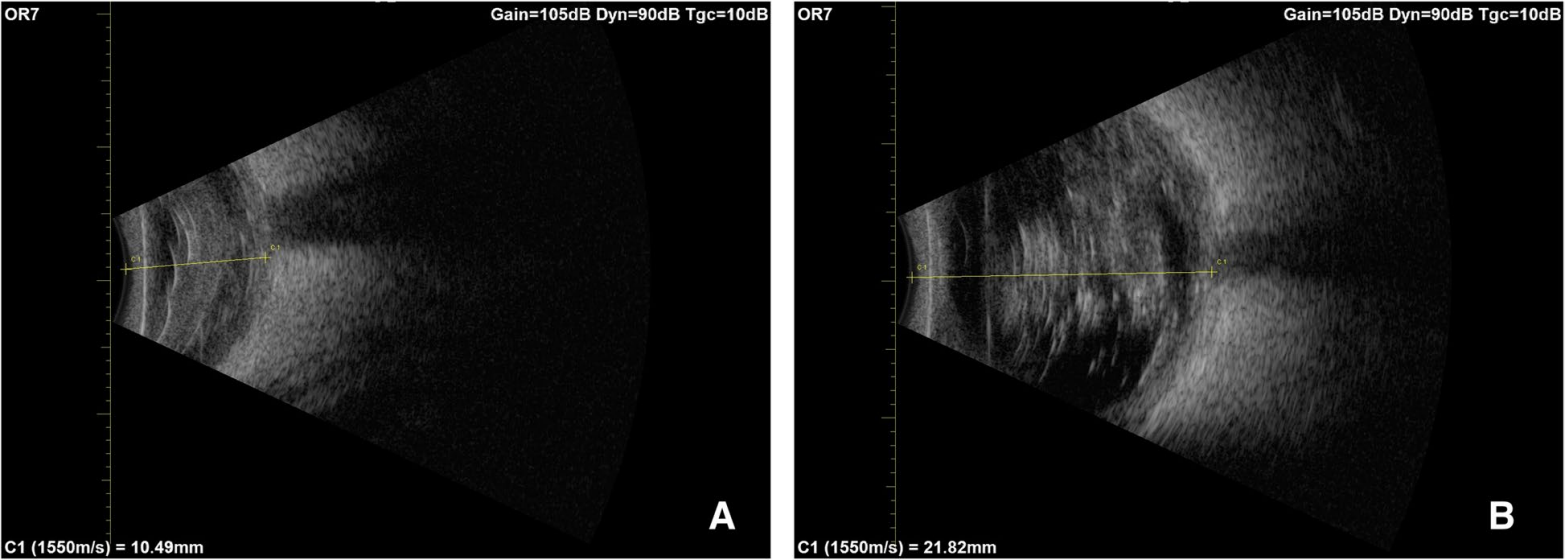
Ultrasound appraisal of a concave eye (**A**) - increase of diameter and sphericity after injection of 3.5 ml of Heptastarch solution (**B**). The posterior border of the lens, granular content of vitreous cavity, the sclera and the optic nerve head can be discerned

On average, the anterior diameter of all the eyes was 14.02 ± 3.82 mm. In a separate analysis, the four convex eyes had a median initial anteroposterior diameter of 19.88 (IQR 19.38–20.05) millimeters, while that of the 16 concave eyes was shorter at 12.59 ± 2.75 mm (see Fig. 2). To establish sphericity, we injected an average of 4.24 ± 2.62 ml of Heptastarch® solution, resulting in a mean ocular diameter of 22.93 ± 1.24 mm. Following volume injection, there was a mean increase of 8.91 mm in the anteroposterior measurement (Fig. 2). However, due to a protocol error, ultrasound imaging after Heptastarch injection was not performed on either eye of the first cadaver head. Nonetheless, imaging of the remaining 18 eyes proceeded without complications.

**Fig. 3:**
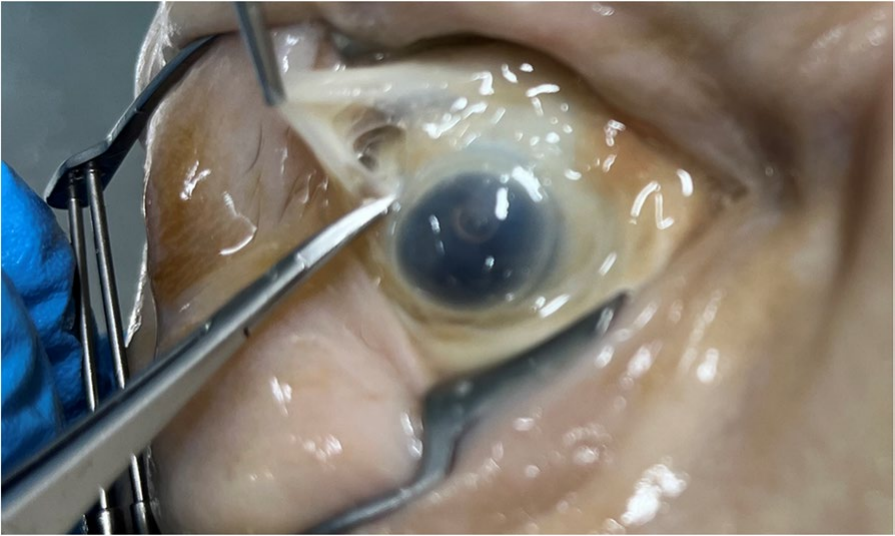
Sub-Tenon block performed in the superotemporal quadrant. Bulbar conjunctiva, Tenon’s fascia and sclera can be discerned but the tissue is friable

Digital assessments indicated an increase in intraocular pressure post-injection, with a gradual rise during the early injection phase and a more rapid elevation toward the end, corroborated by Schioetz tonometry measurements (Table 1). Of note, the injection of Heptastarch® often resulted in very high intra-ocular pressures reaching up to 59.1 mmHg. The initial snip opening of the STBs, involving grasping the conjunctiva and Tenon’s fascia, did not present challenges in any eye, although the embalmed tissue felt more delicate compared to live tissue. Conjunctival color was preserved in pale hues with reduced contrast to the sclera and Tenon’s fascia [14]. Opening the snip typically resulted in larger gaps, measuring 3–8 mm wide, compared to living tissue (see Fig. 3). Nevertheless, we successfully penetrated the conjunctiva-Tenon’s fascia layer, identified the sclera, and positioned the injection cannula correctly behind the eye’s equator in all cases.

**Table 1:** Ocular assessments and measurements

**Case**	**Eye**	**Involuted (Yes/No)**	**Axial length (mm)**		**Volume injected (ml)**	**Approximated Ocular Volume (cm**^**3**^**)**^**a**^	**IOP**^**b**^** (mmHg)**
			**before**	**after**			
OR1	right	No	19.75	NA	1.5	NA	49.8
	left	Yes	10.92	NA	10.0	NA	49.8
OR2	right	Yes	9.05	22.58	5.0	6.03	54.2
	left	Yes	9.01	20.10	8.0	4.25	25.8
OR3	right	No	20.00	24.61	3.0	7.80	42.2
	left	No	20.10	24.11	1.0	7.34	35.8
OR4	right	Yes	10.25	23.24	4.5	6.57	54.2
	left	Yes	15.73	22.74	7.0	6.16	35.8
OR5	right	Yes	16.93	24.17	2.0	7.39	18.5
	left	Yes	14.59	21.77	2.5	5.40	59.1
OR6	right	Yes	17.29	24.01	3.0	7.25	59.1
	left	Yes	11.63	23.36	4.0	6.67	42.2
OR7	right	Yes	10.49	21.82	4.0	5.44	59.1
	left	Yes	13.96	22.69	3.0	6.12	33.0
OR8	right	Yes	9.29	23.82	10.0	7.08	59.1
	left	Yes	12.84	20.56	5.0	4.55	59.1
OR9	right	Yes	13.66	24.00	3.5	7.24	49.8
	left	Yes	11.22	23.54	4.0	6.83	49.8
OR10	right	No	19.00	22.74	0.8	6.16	59.1
	left	Yes	14.61	22.94	3.0	6.32	59.1
*Median*			*13.66*	***23.24***	***4.0***	*6.57*	*49.80*

^a^Based on a mathematical calculation, assuming spherical ocular shape, V = 4/3 π r³

^b^*IOP* Intraocular pressure measured by applanation tonometry with a Schioetz tonometer

**Fig. 4:**
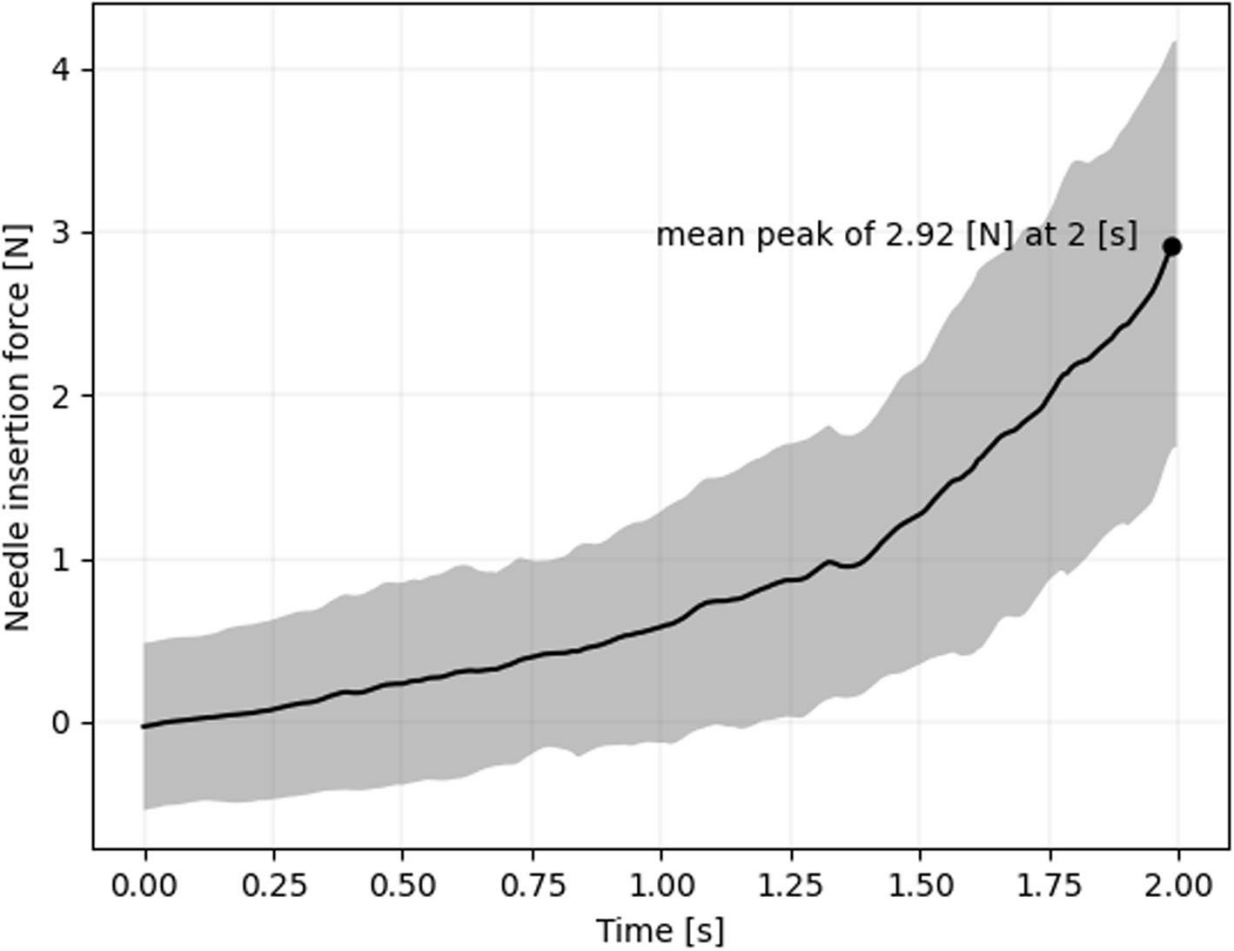
Dynamical insertion forces of the 20 simulated sub-Tenon blocks. Shown are the two seconds leading up to the peak insertion force with the black line as mean and the grey area as the standard deviation

**Fig. 5:**
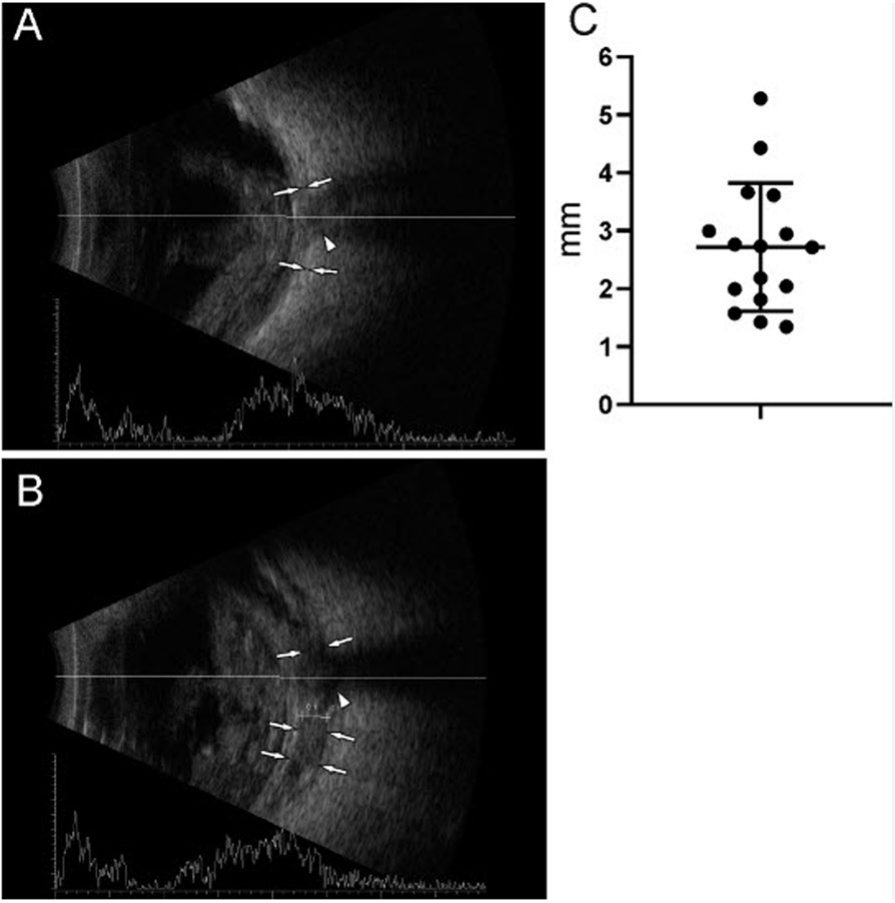
Ultrasound depiction of successful sub-Tenon's blocks with T-sign: A illustrates posterior eye and optic nerve before STB-injection; B shows characteristic "T-sign"ie the fluid depot of injected liquid around optic nerve insertion, averaging ca 3 mm in depth

On average, the peak cannula insertion force was 2.92 ± 1.19 N (Fig. 4) in Thiel cadavers. We observed a significant difference in insertion forces between the supratemporal and inferonasal quadrants (3.27 N vs 2.57 N, respectively, *p* = 0.046). No significant differences were found between individual eyes (see Fig. 4). Measuring insertion forces in isolated pigs eyes, we found a median insertion force of 2.0 N (see Fig. 5).

## Discussion

**Fig. 6:**
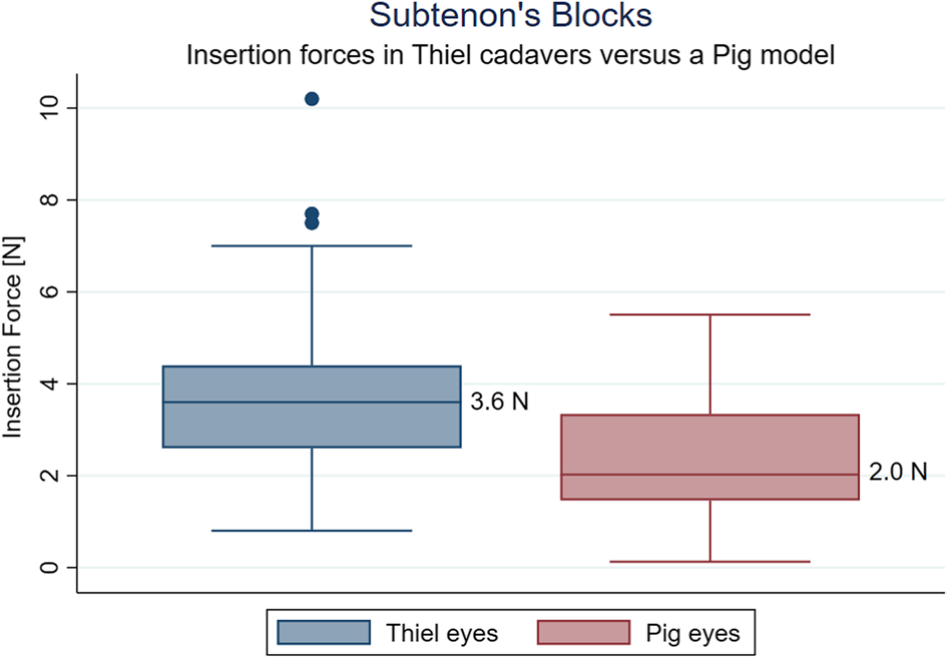
Comparing STB insertion forces between eyes in intact Thiel cadaver heads and isolated pig eyes. Although pig tissue feels harder to penetrate, objective pressures are higher with STB performed on Thiel cadaver eyes. This may well reflect orbital tissue pressure not at play in models of excised eyes

The aim of the study was to assess the suitability of human Thiel cadavers as a training model for sub-Tenon’s (STB) blocks (Fig. 6). Through the evaluation of 40 simulated STBs on 10 Thiel cadaver heads, we observed that while Thiel models provided valuable instructional opportunities, they were with limitations. We noted that a successful sub-Tenon’s block required sufficient visualization of the sclera, followed by positioning of the cannula beyond the equator and familiar haptic feedback to the anaesthetist. This also required the near-physiological sphericity and rigidity of the sclera. All of the 16 eyes in the second cadaver head sample displayed T-signs in ultrasound B-mode interrogation at a mean depth of 2.72 mm (range 1.34 mm–5.28 mm, SD 1.105). This asserts objectively that Thiel cadaver heads are an anatomically valid training model to teach administration of sub-Tenon’s blocks.

Many of the cadaver eyes (16/20) presented in an involuted state after embalming and were concave rather than spherical. The sphericity could be reliably and easily re-established with the injection of Heptastarch® solution into the eye, preferably at the pars plana, approximately 3 to 4 mm from the limbus. This ensured that the relevant ocular tissue layers, that is the conjunctiva, Tenon’s capsule, and sclera, were not affected by the volume injection, while also minimizing its effect on other ocular tissues, such as the choroid and iris-lens-diaphragm. We would argue that achieving physiological intraocular pressures was not a goal of this study, which mostly aimed at re-establishing sphericity and bulbar tonus to allow reproducible application of sub-Tenon’s blocks. While intact under gross inspection, compared to living patients, the bulbar conjunctiva and Tenon’s fascia were friable, harder to grasp by forceps and outlining anatomical layers demanded expertise. Coupled with our finding of collapsed eyes post-embalment, it seems that, compared to muscular and fat tissue in the extremities and trunk, the eyes and orbit are subjected to stronger osmotic forces during embalming. The injection of osmotically effective solutions into the vitreous space of fresh cadavers may mitigate unwanted side effects of the embalming process [15].

Ultrasound sonography was helpful in objectively assessing the sphericity of the eye pre and post Heptastarch® injection while also allowing gross assessment of intraocular structures (choroid, retina, and vitreous body). As expected, the intraocular layers appeared somewhat disorganized, however, landmarks such as the optic nerve head and scleral wall could be identified with a high level of confidence. This may be of interest when attempting to model ocular complications of regional anaesthesia like scleral perforation from peribulbar blocks or retrobulbar haemorrhage; indeed, there are reports in the literature regarding this topic [16].

Our study’s force measurements provide quantifiable data for comparing cadaver models to living surgical patients, especially in the hands of experienced clinicians. However, their true value will be fully realized when similar measurements are obtained from living patients, allowing for direct comparison and validation. We observed a notable discrepancy in insertion forces between the superotemporal and inferonasal quadrants, although the reason for this difference remains unclear. Despite this, the documented insertion forces from our study will serve as a valuable reference point for future investigations and contribute to advancing understanding in this area.

We used a 10 MHz probe for our ultrasound imaging, which provided excellent images of the posterior eye wall and intraocular tissues. However, ultrasound resolution did not permit discerning anterior tissue layers involved in the sub-Tenon’s block. It remains to be seen whether this connective tissue can be imaged reproducibly with high-resolution 50–60 MHz ultrasound-bio-microscopy in Thiel cadaver eyes and consequentially compared with healthy individuals. This has previously been done in sub-Tenon’s needling in glaucoma therapy [17]. In future studies, ultra-sonographic testing for the emergence of T-signs behind the bulb would be an advantage, completing the anterior appraisal of chemosis and fluid run-off [18].

Insertion forces have been assessed in various areas of regional anaesthesia to mitigate nerve damage, as noted in previous literature, including studies on epidural training [7]. The significance of assessing insertion forces lies in understanding the balance between achieving ideal tissue characteristics and maintaining anatomical fidelity. This balance is essential for ensuring safe and effective procedures while also providing valuable learning experiences that prepare clinicians to manage potential complications effectively. In our case, the efforts to achieve satisfactory sphericity, low pressure, and high friability of exceedingly soft tissues must be weighed against the anatomical fidelity and the instructive possibility of feeling catastrophic complications such as bulbar perforation, optic nerve injection or intramuscular injection [15]. Insertion forces in our Thiel model were higher than in fresh excised pig eyes. The most likely explanation for this difference seems to lie in the orbit’s tissue influencing insertion forces. This is all the more plausible given that pig tissue feels coarser while performing STB. It remains to be established in the future where insertion forces in living patients of all ages come to lie and which physiological factors determine them.

Our feasibility study has several limitations. Firstly, the lack of functional assessment poses a significant limitation. While the study evaluated the anatomical aspects of simulated sub-Tenon’s (STB) blocks using Thiel cadavers, it did not assess the functional outcomes of these procedures, such as the efficacy of anaesthesia or the spread of injected fluids. The inability to monitor fluid spread during the mock STB procedures represents a notable limitation.

The observed discrepancy in intraocular pressure (IOP) readings, particularly in the ‘convex’ eyes, raises a potential concern regarding the accuracy of tonometer measurements. Given the soft and involuted nature of most cadaver eyes, it is reasonable to expect lower IOP levels. Additionally, the low axial length (AL) measurements in these eyes further suggest the possibility of low IOP. The use of traditional ‘finger IOP measurement’ by an experienced ophthalmologist could have provided more reliable results, as other tonometers may inaccurately measure IOP in cadaveric eyes. While the exact IOP levels are not critical to the study’s objectives, our main objective was to establish bulbar sphericity. As a minor drawback we had to accept high intraocular pressures in these cadaver eyes.

The study’s small sample size is another important limitation. With only 40 simulated STBs, the sample size may not be sufficient to draw robust conclusions. Lastly, the study lacked a clear definition of success for STB blocks conducted on cadavers. As analgesic properties cannot be evaluated in cadavers, defining success based solely on anatomical criteria may not fully capture the clinical relevance of STB procedures. Assessing ultrasound T-signs as surrogate, we believe, only in part compensates this fundamental limitation. A more comprehensive definition of success, incorporating both anatomical and functional outcomes where possible, would enhance the interpretability and applicability of the study findings to clinical practice.

However, the study stands out for its innovative investigation, addressing a gap in existing research. Through a methodically rigorous approach, we evaluated various aspects essential for successful STB procedures, including anatomical characteristics and tissue properties. Its findings not only provide valuable insights into the feasibility and effectiveness of using Thiel cadavers for STB block training but also have practical implications for enhancing medical education and training in regional anesthesia.

Overall, our experiments demonstrated that Thiel cadaver heads provide sufficient potential as anatomically valid models for ocular regional anaesthesia [19] with macroscopically maintained connective tissue layers, characteristic insertion forces and the presence of ultrasound T-signs after injection of fluid into the sub-Tenon’s space.

### Supplementary Information

Supplementary Material 1.

Supplementary Material 2.

Supplementary Material 3.

Supplementary Material 4.

## Data Availability

Data is provided within the manuscript or supplementary information files.

## Abbreviations

IOP: Intraocular pressure
SD: Standard Deviation
STB: Sub-Tenon’s block
US: Ultrasound
